# Outcomes of Follow-up Imaging After Pediatric Spinal Trauma Confirmed With Magnetic Resonance Imaging

**DOI:** 10.1097/BPO.0000000000002615

**Published:** 2024-01-15

**Authors:** Aapo Sirén, Johanna Syvänen, Mikko Nyman, Kimmo Mattila, Jussi Hirvonen

**Affiliations:** Departments of * Radiology; † Pediatric Orthopedic Surgery, University of Turku and Turku University Hospital, Turku; ‡ Department of Radiology, Medical Imaging Center, Tampere University and Tampere University Hospital, Tampere, Finland

**Keywords:** follow-up imaging, magnetic resonance imaging, pediatric, spine, trauma

## Abstract

**Background:**

Imaging plays a crucial role in the diagnostic workup of pediatric spinal trauma. Computed tomography and conventional radiographs are widely used as the primary imaging methods. Magnetic resonance imaging (MRI) is a radiation-free alternative with high sensitivity for bony and soft tissue injuries. There is no consensus on the optimal use of follow-up imaging in pediatric spinal trauma without immediate surgical treatment, especially if the injury is primarily confirmed with MRI. This study aimed to assess the diagnostic value of follow-up imaging after MRI-confirmed spinal trauma in children.

**Methods:**

The medical records and the imaging data of children and adolescents with emergency spinal MRI and follow-up imaging over 8 years were retrospectively reviewed. The primary study outcome was the outcome of follow-up imaging and its effect on management.

**Results:**

The study population consisted of 127 patients. The follow-up imaging did not alter the management in any patient with presumably stable injury in emergency MRI. Short-term follow-up imaging showed no clinically significant progression in thoracolumbar compression fractures. Flexion-extension radiographs had no additional value in cases with stable cervical spinal injury on emergency MRI.

**Conclusions:**

The clinical utility of short-term follow-up imaging is low in children with stable spinal injury on emergency MRI.

**Level of evidence:**

Level III—retrospective observational study.

Pediatric spinal trauma is uncommon. Spinal fractures are estimated to represent 1% to 3% of all pediatric fractures,^1^ and in a large Finnish registry-based study, the annual incidence of spinal trauma requiring hospitalization was 1 per 15,000 children.^2^ The exact incidence of spinal trauma without needing surgical care is unknown in the pediatric population. Because spinal injuries are rare but potentially fatal, the most suitable primary imaging method is still under discussion. The role of imaging is to detect potentially unstable injuries demanding surgery or brace immobilization. The most common potentially unstable injuries include posterior ligamentous complex (PLC) or anterior tension band injury, burst fractures, and disruption of the ligaments stabilizing the occipitocervical junction.^3,4^ Conventional radiographs and computed tomography (CT) are widely recommended for first-line imaging,^3,5^ but magnetic resonance imaging (MRI) is more sensitive.^6–8^ Important stabilizing ligaments of the spine are usually not visible on CT or conventional radiographs. Potential injuries in these structures must be assessed with secondary findings on bony structures, such as avulsion fractures and vertebral misalignment. MRI is superior in visualizing the ligaments and comparable to CT in detecting bony injuries.^9,10^ A recent retrospective analysis found MRI safe and 100% sensitive for unstable injuries in the pediatric population with suspected cervical spine trauma.^11^ The most significant drawbacks of the MRI include the need for anesthesia in younger or restless children, higher cost per examination, and longer scanning time compared with CT or conventional radiographs. Despite the higher sensitivity in ligamentous injuries, MRI does not necessarily yield additional value in the treatment of pediatric spinal trauma,^12–14^ but its major advantage over CT is the lack of ionizing radiation.

Knowledge about the need and implementation of follow-up imaging, especially short-term follow-up imaging in conservatively treated pediatric patients with spinal trauma, is scarce; it is mainly based on expert opinions and studies in which the primary spine clearance has been made with plain radiographs.^15^ The diagnostic yield of follow-up imaging after spinal trauma with MRI as emergency imaging is poorly known. The clinical significance of stable injuries on MRI and the need for follow-up imaging has not been studied in the cervical spine. The utility of short-term radiologic follow-up in minor compression fractures regarding the thoracolumbar spine is also unclear. The risk for persistent postural malalignment in pediatric compression fractures seems to be associated with older age and Risser grade 2 or higher,^16,17^ albeit the average functional outcome appears to be favorable irrespective of the age and radiographic result.^16,18,19^

It has been shown that MRI can safely be used as a first-line imaging modality for low-impact pediatric spinal trauma.^20^ The purpose of the current study was to examine the additional value of follow-up imaging after pediatric spinal trauma initially confirmed on MRI. Our hypothesis and practical rationale were that by leveraging the excellent accuracy of MRI in detecting and excluding spinal injuries, the amount of follow-up imaging of patients primarily scanned with emergency MRI could be reduced.

## METHODS

We retrospectively reviewed the charts of pediatric patients (n=634) who had undergone an emergency spinal MRI at our institution between April 1, 2013, and August 31, 2021. We operate an emergency radiology department at a tertiary care referral center for 470,000 people. The inclusion criteria for the study were (1) emergency spinal MRI due to acute trauma, (2) age under 18, and (3) follow-up imaging after the initial hospitalization period. The patients who underwent surgery after the first-line imaging studies (n=6) were excluded. The study is entirely retrospective; hence, the choices regarding primary imaging, therapy, and follow-up imaging reflect the discretion of the responsible physician (pediatric orthopedic surgeon).

The emergency MRI scans were performed with a Philips Ingenia 3-T system and Philips dStream coils (Philips Healthcare, Best, the Netherlands). The standard MRI protocol included sagittal T1-weighted, sagittal and axial T2-weighted, sagittal and coronal short tau inversion recovery (STIR), sagittal diffusion-weighted, and sagittal gradient-echo T2*-weighted. In selected cases, dedicated imaging of the level C0-C2 was performed with a small field of view proton density-weighted and T2-weighted series. The follow-up MRIs, CTs, and conventional radiographs were obtained using devices by various vendors. A low-dose upright bi-plane slot-scanning (The EOS System, ATEC Spine Inc., Carlsbad, CA) was often used in follow-up with conventional radiograph imaging. Emergency MRI scans were read by a fellowship-trained musculoskeletal radiologist, neuroradiologist, or emergency radiologist with at least seven years of experience in radiology. A fellowship-trained neuroradiologist or pediatric radiologist with at least 7 years of experience in radiology read the follow-up images. This study is a retrospective chart review, and the analysis is based on original radiology reports. We were interested in the follow-up imaging’s impact on the patient treatment, and therefore, we did not perform retrospective image interpretation. We could not include the image review of the responsible pediatric orthopedic surgeon in our analysis because the surgeons’ conclusions about emergency MRI or follow-up imaging were not systematically recorded in the patient charts.

Information about the imaging studies, radiology reports, treatment, and patient outcomes was extracted from the radiology information system, picture archiving and communication system, and clinical medical records.

This study aimed to assess the diagnostic yield of follow-up imaging after MRI-confirmed pediatric spinal trauma. We examined the impact of follow-up imaging on treatment plans and the progression of the traumatic findings in the follow-up imaging. A particular focus was on 2 groups: (1) the patients with cervical spine trauma and flexion-extension (FE) follow-up imaging and (2) the patients with one or multiple thoracolumbar vertebral compression fractures with height loss no >30% in any vertebral body in primary imaging.

The MRI findings strongly indicating instability were defined as follows: PLC or anterior tension band disruption, burst fractures, and disruption of the ligaments stabilizing the occipitocervical junction.

The results are expressed as the number of cases (n), percentages, means, and SDs. Ordinal variables were compared with the χ^2^ test. *P*-values <0.05 were considered statistically significant. We performed the statistical analyses with IBM SPSS Statistic for Mac (version 28, IBM Corporation, Armonk, NY).

The hospital district board’s permission for the study was obtained, but the institutional review board’s approval or written patient consent was not needed for the retrospective study.

## RESULTS

We found a total of 133 patients meeting the inclusion criteria. After excluding 6 patients who underwent immediate surgery, the final study sample included 127 patients. Table 1 presents the essential demographic characteristics, injury mechanisms, additional emergency imaging, and an overview of the follow-up imaging studies among the study sample. The findings of the primary MRI are presented in Table 2.

**TABLE 1:** The Overall Characteristics of the Study Population and Imaging Studies

Population descriptives	
No. cases	127
Age, mean (SD), range	11.3 (3.2), 2–17
Female, n (%)	55 (43.3)
Mechanism of injury	n (%)
Trampoline	31 (24.4)
Sports	28 (22.0)
Fall	27 (21.3)
Traffic	25 (19.7)
Horseback riding	8 (6.3)
Violence by another child	4 (3.1)
Diving	2 (1.6)
Other	2 (1.6)
Additional primary imaging	n (%)
Computed tomography	46 (36.2)
Conventional radiographs	12 (9.4)
Follow-up imaging overview	Mean (SD) range
First follow-up imaging (d)	24 (21) 1–171*
Last follow-up imaging (d)	58 (177) 1–2011*
No. follow-up imaging	1.6 (0.1) 1–5

* The single patient with extraordinary long follow-up time was scanned after 171 days because of persisting and exacerbating spinal symptoms and again in 2011 days after the initial trauma. No follow-up imaging was planned in the first place.

**TABLE 2:** The General Characteristics of Findings in the Emergency Magnetic Resonance Imaging

General characterization of primary findings on MRI	n (%)
Bony injury only	60 (47.2)
Bony and ligamentous injury	30 (23.6)
Ligamentous injury only	25 (19.7)
Other*	3 (2.4)
No acute findings on MRI	9 (7.1)
Injured levels on primary MRI	
Cervical spine	40 (31.5)
Thoracic spine	32 (25.2)
Lumbosacral spine	13 (10.3)
Multiple levels	33 (26.0)
None	9 (7.1)

* Intervertebral disk protrusion, muscle/soft tissue injury, and retroclival hematoma without observable ligamentous injury.

MRI indicates magnetic resonance imaging.

### Patients With Only Thoracolumbar Contusions or Compressions

We found 42 patients with one or more thoracolumbar vertebral contusions or compression fractures with ≤30% vertebral height loss (Table 3). The compression fractures were unchanged in the follow-up imaging in 41 (98%) cases. In one patient (2%), a slight progression was seen. This 14-year-old patient had a compression fracture in the vertebral body of the 12th thoracic vertebra. The height loss increased from 5% to 10% at 2-week conventional radiographs but remained stable in later studies. The latest spinal imaging of this patient was a lumbar MRI 10 months after the injury due to nonspecific lower back pain. None of the short-term follow-up images of uncomplicated thoracolumbar compressions led to any additional treatment.

**FIGURE 1:**
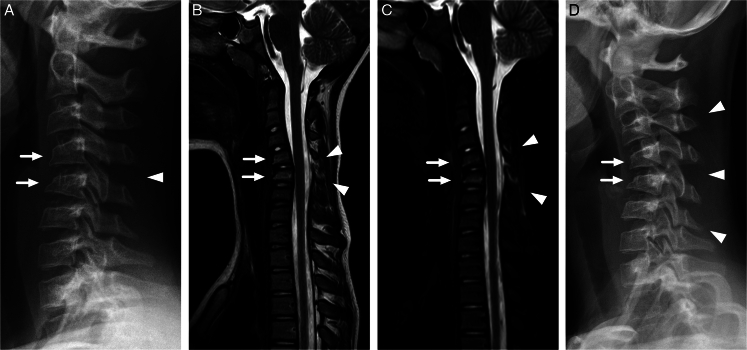
Posterior ligamentous complex injury and vertebral compression fractures in a 10-year-old girl after a fall. A, Primary lateral plain radiograph from an outside hospital with vertebral compression fractures (arrows), interspinous distance widening (arrowhead), and abnormal kyphosis. Sagittal T2-weighted series (B) and Sagittal short tau inversion recovery series from emergency magnetic resonance imaging show vertebral compression fractures (arrows) and posterior ligamentous complex injury with abnormal kyphosis (C). A facet joint capsule injury was also present. D, Follow-up lateral plain radiograph after a 1-week attempt of conservative treatment with a rigid cervical collar demonstrates the progression of the traumatic kyphotic malalignment. The surgical fixation was performed.

**TABLE 3:** The Patients With Only Thoracolumbar Vertebral Contusion or Compression ≤30%

No. cases	42
Age, years (SD)	10.4 (3.0)
Female n (%)	19 (45)
Maximal compression, % of the vertebral height, mean (SD), range	10 (8), 0–30
No. injured vertebrae, mean (SD), range	3.2 (2.0), 1–7
Thoracic spine injury, n (%)	28 (67)
Lumbar spine injury, n (%)	8 (19)
Combined thoracic and lumbar spine injury, n (%)	6 (14)
Immobilization with a stiff collar or brace, n (%)*	28 (67)
Follow-up conventional radiographs (including flexion-extension imaging)†	42 (100)
Follow-up CT n (%)	1 (2)
Follow-up MRI n (%)	2 (5)
First/last follow-up imaging, mean days (SD)	20 (14)/40 (13)
Number of follow-up imaging per case, n (SD)	1.6 (0.6)
Progression in imaging findings on follow-up imaging, n (%)	1 (2)
Additional treatment based on follow-up imaging, n (%)	0

* The stiff collar was used in cases with upper thoracic spine compressions.

† The flexion-extension radiographs were used in one case with upper thoracic spine compressions.

CT indicates computed tomography; MRI, magnetic resonance imaging.

### Patients With Cervical Spine Injury and FE Follow-up Imaging

Our study population had 54 patients with cervical spine injury and FE follow-up imaging (Table 4). Of these, 36 were scanned with conventional radiographs, 6 with MRI, and 10 with both. None of the patients without findings strongly indicative of instability in the emergency MRI had signs of instability in the follow-up FE imaging. In contrast, instability was seen on FE imaging in 6/14 (43%) patients with presumed instability on emergency MRI (*P*<0.001). Five patients were referred to delayed surgical treatment after persistent instability, concord with the emergency MRI findings. The patients with MRI findings highly suggesting instability are presented in Supplemental digital content 1, http://links.lww.com/BPO/A702.

**TABLE 4:** The Patients With Flexion-extension Follow-up Imaging

	Patients with primary MRI findings suggesting instability*	Patients without primary MRI findings suggesting instability	*P*
Findings suggestive of instability on follow-up flexion-extension imaging	6/14	0/39	<0.001 (χ^2^ 18.848)
Additional treatment based on follow-up flexion-extension imaging	5/14	0/39†	0.004 (χ^2^ 11.277)

* Posterior ligament complex injury or potentially unstable fracture.

† In one case, the collar treatment was abandoned after the flexion-extension imaging showing no signs of instability.

MRI indicates magnetic resonance imaging.

Further, we specifically assessed the value of the FE MRI in follow-up imaging. None of the 16 FE MRI examinations revealed findings not seen in the emergency MRI and static follow-up MRI. Additional FE MRI gave no further information compared with conventional FE imaging. Achieving a satisfactory range of motion in FE MRI was more challenging than in FE radiographs. The policy about when the FE imaging was requested with radiographs or MRI was not evident but instead based on the preference of individual pediatric orthopedic surgeons.

### Other Patients With New Imaging Findings on Follow-up Imaging

Only 2/127 (2%) patients’ follow-up imaging revealed trauma findings, suspected or confirmed, that were not primarily seen in the emergency MRI. The first of these patients had no findings on the emergency MRI but was suspected of PLC injury on the follow-up FE radiographs. The new MRI still did not show edema, ligamentous disruption, malalignment, or any other finding suggestive of trauma. The second patient was found to have primarily missed injuries in the apical ligament and the tectorial membrane in a brain MRI 2 weeks after the injury. The conservative treatment was, however, successfully continued as planned. The patient was known to have idiopathic thoracolumbar scoliosis that was later operated on and followed up by a pediatric orthopedic surgeon for four years after the injury. Symptoms related to the cervical spine trauma did not occur during the clinical follow-up period.

In addition to the cases described under the FE imaging, 1 patient required surgery due to the follow-up imaging findings. This patient’s emergency MRI findings were compression fractures on C4 and C5, bilateral C4/5 facet joint capsule injuries, partial interspinous ligament tear, and ligamentum flavum detachment from the vertebral arches on levels C4-C5. There was no unequivocal ligamentous discontinuity, but slight kyphotic malalignment without spondylolisthesis was seen. As the kyphosis significantly progressed at the 1-week plain radiograph follow-up imaging without FE views, the posterolateral instrumented fusion was performed (Fig. 1).

## DISCUSSION

Findings from 127 pediatric patients suggested a low added value of short-term follow-up imaging after primarily MRI-confirmed spinal trauma without findings strongly indicating instability. Therefore, follow-up imaging may not be warranted after a stable injury has been detected primarily by MRI.

The diagnostic accuracy of emergency MRI was excellent—none of the follow-up imaging revealed primarily unnoticed findings that would lead to a change in the treatment plan. The injuries with delayed operative treatment due to follow-up imaging findings were considered potentially unstable (Fig. 1) but possibly stabilizing with a brace during the follow-up. In case of potentially unstable injury without immediate surgery, follow-up imaging is unquestionably needed. The follow-up imaging did not alter the initial treatment protocol of any patient without primary MRI findings strongly suggesting instability. These findings may increase the reliance on emergency MRI in clinical decision-making and help avoid redundant follow-up imaging. Even if the follow-up imaging of spinal injuries without unstable features might seem redundant in the first place, it is not uncommon in clinical practice. Nonmedical reasons like fear of malpractice claims or parental anxiety can lead to additional imaging. The knowledge about the accuracy of emergency spinal MRI in injuries with primarily benign appearance is also sparser than the better-known accuracy of CT.

Of the 42 patients with 1 or more thoracolumbar vertebral compression fractures with ≤30% height loss and no additional injuries, 1 patient had a slight progression in the loss of vertebral height. The treatment plan was not altered (Table 3) in any case. Especially, older children and adolescents are known to have an increased risk of post-traumatic scoliosis.^17,18^ Our study suggests that if ligamentous injuries, burst fractures, and other potential complicating factors are excluded with emergency MRI, the short-term follow-up imaging of the mildly compressed vertebrae with a height loss of ≤30% might not be necessary. The long-term radiologic outcome of these injuries is beyond the scope of this article. More study is needed to understand better the factors that may lead to clinically significant post-traumatic scoliosis or kyphosis in the long term.

If the emergency MRI findings did not indicate unstable injury, we found the overall diagnostic yield of the FE imaging low (Table 4). That concords with the recent findings by Zhang et al.^21^ Considering the FE imaging technique, the additional value of dynamic MR imaging of the cervical spine was minor compared with standard MRI and conventional FE radiographs. A satisfactory range of motion in FE imaging was more challenging to achieve in MRI than in plain radiographs. When FE imaging is needed, low-dose techniques like the EOS system can be used for dynamic radiographs.^22^ However, if both FE imaging and structural MRI are required, the FE MRI might be a good option for a cooperative child or adolescent. Combined imaging does not bring additional advantages.

Ionizing radiation increases future cancer risk, especially among the vulnerable pediatric population.^23,24^ At the population level, avoiding unnecessary examinations is the most efficient way to reduce radiation exposure. Our results might help to reduce follow-up imaging using ionizing radiation and strengthen the reliance on the emergency spinal MRI as a reliable but radiation-free alternative to the CT. In addition to reducing radiation exposure, refraining from unnecessary follow-up imaging might help to balance the higher direct cost of emergency MRI. The favorable effect of cervical emergency MRI on overall treatment cost has already been proposed due to shorter intensive care unit stays.^25^ The need for fewer follow-up imaging might further contribute to reducing the costs.

Our study has certain limitations, the most obvious being its retrospective nature and inherent biases. The study is a single-center study with a small sample size, which diminishes the generalizability of the results. Nevertheless, by employing the availability of emergency MRI in our institution, we can provide results that may be clinically valuable and worth examining in a larger sample, especially considering the limited knowledge of the need for follow-up imaging after a spinal emergency MRI. One important limitation is the lack of a control group without follow-up imaging after an emergency MRI. However, in our previous work, we found no missed injuries requiring surgical interventions after MRI as a first-line imaging in low-impact pediatric spinal trauma.^20^ Therefore, we are confident that no patients with potentially unstable injuries would have left without adequate follow-up. Because of the excellent sensitivity of MRI,^7^ the follow-up imaging may have been applied to patients whose injuries would not have been detected on CT or conventional radiographs and who would not have been followed up without an emergency MRI. Still, this would not bias our main message—the low diagnostic yield of the short-term follow-up imaging after MRI-confirmed stable spinal trauma in children and adolescents.

In conclusion, we found low diagnostic yield and limited clinical utility of follow-up imaging after stable injuries on emergency MRI in children and adolescents. With the previous findings on the safety and accuracy of emergency MRI for pediatric spinal trauma, the current findings might increase the reliance on emergency MRI in clinical decision-making.

## Supplementary Material
