# Supplementary material for: Outcomes of Follow-up Imaging After Pediatric Spinal Trauma Confirmed With Magnetic Resonance Imaging
Source: J Pediatr Orthop. 2024 Jan 15;44(4):e329–34. doi: 10.1097/BPO.0000000000002615 (PMC10913857; doi:10.1097/BPO.0000000000002615)
Supplement: Supplementary file 1 [file bpo-44-e329-s001.docx]

**Supplementary material: The patients with emergency magnetic resonance imaging findings highly concerning for unstable injury**

| Age | Injury mechanism | Emergency MRI findings | Initial treatment | Follow-up imaging | Follow-up findings | Treatment after follow-up imaging |
| --- | --- | --- | --- | --- | --- | --- |
| 3 | Fall | Partial flavum and interspinous ligament tears on level C3/4 | Rigid collar for 6 weeks | MRI and FE radiographs at 6 weeks | MRI: edema had disappeared, no ligamentous discontinuity. FE radiographs: negative | No additional therapy, successful conservative treatment |
| 6 | Trampoline | Flavum tear on level C5/6, interspinous ligament tear on levels C4-6, mild compression fractures on Th1-Th5 | Rigid collar for 8 weeks | FE radiographs at 6 weeks | No malalignment in FE radiographs, compression on Th1 as on MRI | No additional therapy, successful conservative treatment |
| 8 | Trampoline | Partial flavum tear on level C7/Th1, interspinous ligament tear on level C7-Th2, compression fractures on Th1-Th3 | Rigid collar for 6 weeks | Plain radiographs at 1 and 6 weeks, the latter combined with FE radiographs | No malignment, compression fractures as on MRI | No additional therapy, successful conservative treatment |
| 10 | Trampoline | Flavum, interspinous-, and nuchae ligament tears on level C2/3, edema on occipitocervical muscles, compression fractures on Th2-Th3 | Rigid collar for 6 weeks | FE radiographs at 2 weeks, cervical MRI at 6 weeks | FE radiographs: kyphosis and instability on level C2/3. MRI: progression in kyphosis and malalignment, traumatic edema has diminished | Surgery; Posterolateral instrumented fusion C2-3 |
| 10 | Trampoline | Flavum tear, interspinous ligament tear and right-sided facet joint capsule injury on level C2/3 | Rigid collar for 6 weeks | FE radiographs at 2 and 8 weeks, FE MRI at 6 months | FE radiographs: mild instability on level C2/3 at both 2 and 6 weeks. FE MRI: no instability, no edema, no ligament discontinuity. | At 2 weeks, the collar treatment was extended for 8 weeks in total. At 8 weeks, the collar was removed  as the patient was symptom-free. No further treatment at 6 months. Successful conservative treatment. |
| 10 | Trampoline | Flavum ligament tear on level C1/2, compression fracture on Th2 | Ridig collar for 6 weeks | Cervical FE radiographs at 6 weeks | No malignment, compression fracture as on MRI | No additional therapy, successful conservative treatment |
| 11 | Trampoline | Flavum: partial tear on level C4/5 and major tear on level C6/7, interspinous ligament tear on C4-C7, facet joint capsule injury on C6/7, interspinous space widening on C4/5 and C6/7, substantial posterior soft tissue edema on levels C0-C7, compression fractures on C7 and Th2 | Rigid collar for 6 weeks | Plain radiographs at 2 weeks, FE radiographs at 6 weeks, FE MRI at 12 weeks, FE radiographs at 18 weeks | 2 and 6 weeks: interspinous space widening, facet joint and vertebral body malalignment on level C6/7. Compressions were unchanged. 12 weeks: As on previous follow-up imaging. No segmental instability on FE imaging. 18 weeks: The previous findings persisted. Poor range of motion at this level C6/7. | At first, the collar treatment was extended for 12 weeks in total. After the FE radiographs at 18 weeks, the patient was operated on (anterior fusion with plate and cage C6-7) |
| 11 | Trampoline | Partial flavum tear, interspinous- and nuchae ligament tears on level C6/7. Mild edema on the level C1/2 posterior to flavum. Subcutaneous hematoma and substantial edema at the posterior soft tissues on the level C4-7. | Rigid collar for 6 weeks | Plain radiographs at 1 and 2 weeks, FE MRI at 6 weeks | Plain radiographs at 1 and 2 weeks: widened interspinous distance on the level C6/7. FE MRI: Widened interspinous distance increasing in flexion. Soft tissue edema has diminished. | Surgery after 6 weeks; posterolateral instrumented fusion C5-Th1 |
| 11 | Trampoline | Flavum- and interspinous ligament tear on the level C4/5, compression fractures on Th2-Th5 | Rigid collar for 6 weeks | Plain radiographs at 2 and 6 weeks, the latter with FE images | No malalignment or instability, compression fractures as on MRI | No additional therapy, successful conservative treatment |
| 11 | Trampoline | Partial tears on flavum and interspinous ligaments, 2 mm spondylolisthesis and facet joint subluxation on the level C5/6 | Rigid collar for 6 weeks | FE radiographs at 6 weeks | No malalignment or instability | No additional therapy, successful conservative treatment |
| 12 | Diving | Flavum-, and interspinous ligament tears, kyphosis and 2 mm spondylolisthesis and facet joint subluxation on the level C5/6. Mild compression fracture on C5. | Rigid collar for 8 weeks | FE radiographs at 8 weeks, MRI at 10 weeks | FE radiographs: 4 mm facet joint subluxation on flexion. MRI: 1 mm spondylolisthesis, 2 mm facet joint subluxation. Edema has diminished. Compression fracture unchanged. | No additional therapy, successful conservative treatment. The rigid collar was removed after 8 weeks. |
| 13 | Horseback riding | Flavum- and interspinous ligament tears and right-sided facet joint capsule injury on the level C4/5. Edema at the spinous processes of C4 and C5, no dislocated fracture | Rigid collar for 6 weeks | Plain radiographs at 2 weeks, FE MRI at 6 weeks | Plain radiographs: Mild kyphosis at the level C4/5. FE MRI: No malignment or instability. Soft tissue edema has diminished, no ligamentous discontinuity. The spinous process of the C4 is still edematic, consistent with a healing fracture (no dislocation). | At 6 weeks, the collar treatment was extended for 8 weeks total due to the still edematic spinous process. Successful conservative treatment. |
| 14 | Motor vehicle accident | Fracture of the posterior arch of the atlas and partial tear at the atlantooccipital membrane | Rigid collar for 8 weeks | Plain and FE radiographs at 8 weeks, focused CT later on the same day to assess the fracture healing process further. | Plain and FE radiographs: No malalignment or instability. The atlas fracture is better seen in FE radiographs. CT: Partial bridging callus at the fracture, no dislocation. | The collar treatment was extended to 12 weeks in total to allow fracture healing. Successful conservative treatment. |
| 17 | Motorcycle accident | Hangman’s fracture, left atlantoaxial joint capsule injury, vertebral contusion on C7 | Rigid collar for 6 weeks | Plain radiographs at 2 and 6 weeks | 2 weeks: 2 mm spondylolisthesis on the level C2-3, the fractures as on MRI. 6 weeks: 2 mm spondylolisthesis on the level C2-3, no facet joint incongruence, fractures not visible anymore | No additional therapy, successful conservative treatment |
